# Realities of using self-administered smartphone surveys to solve sustainability challenges

**DOI:** 10.1057/s41599-025-05305-w

**Published:** 2025-07-19

**Authors:** Amy R. Lewis, Simon Willcock, Ana Casas, Beata Kupiec-Teahan, José Mendoza Sanchez, Fiona Anciano, Dani J. Barrington, Mmeli Dube, Paul Hutchings, Caroline Karani, Arturo Llaxacondor, Hellen López, Anna L. Mdee, Keosothea Nou, Alesia D. Ofori, Joy N. Riungu, Kory C. Russel, Md Ehsanul Haque Tamal, Alison H. Parker, Andrew R. Bell

**Affiliations:** 1 Bangor University, Bangor, UK; 2 Rothamsted Research, Harpenden, UK; 3 Cranfield University, Cranfield, UK; 4 Pontifical Catholic University of Peru, Lima, Peru; 5 Institute of Peruvian Studies, Lima, Peru; 6 University of the Western Cape, Cape Town, South Africa; 7 University of Western Australia, Perth, WA Australia; 8 University of Leeds, Leeds, UK; 9 Meru University of Science and Technology, Meru, Kenya; 10 Sanima, Lima, Peru; 11 Prek Leap National Institute of Agriculture, Phnom Penh, Cambodia; 12 University of Oregon, Eugene, OR USA; 13 Murdoch University, Perth, WA Australia; 14 Cornell University, Ithaca, NY USA

**Keywords:** Environmental studies, Geography, Sociology

## Abstract

To fill data gaps in human-environment systems, especially in difficult-to-access locations, novel tools are needed to collect (near) real-time data from diverse populations across the globe. Here we discuss the practicalities, constraints, and lessons learnt from six field studies using high spatial and temporal smartphone surveys in six different countries. We suggest that high spatiotemporal, self-administered smartphone surveys will produce novel insights into human behaviour, attitudes, and socio-economic characteristics that, when matched with high spatiotemporal resolution environmental data (e.g., from remote sensing), can be used to address sustainability challenges for global communities. Furthermore, we highlight the need for continuous refinement and improvement in future developments to enhance the efficacy of this methodology. By sharing the practical implications and constraints associated with smartphone surveys, this article contributes to the evolving landscape of data collection methods.

## Introduction

Sustainable development is a pressing and complex issue that requires a comprehensive understanding of human-environment systems (Glaser et al., 2012; Kabisch et al., 2015; Dearing et al., 2006; Arias-Maldonado, 2015; Soga and Gaston, 2020). Although the United Nations Sustainable Development Goals (SDGs; UN, 2015) provide a framework for addressing this challenge, achieving and evaluating successful delivery of these goals rely on the availability and accuracy of data (Lu et al., 2015). Despite advances in data collection, there remains a critical data gap on the interactions between people and the environment, which hinders the development of effective policies across numerous disciplines (e.g., urban ecology, human ecology, and sociology; Glaser et al., 2012; Kabisch et al., 2015; Dearing et al., 2006; Arias-Maldonado, 2015; Soga and Gaston, 2020) and challenges (such as climate change, inequality, and poverty; Scharlemann et al., 2020).

A major impediment to advances in the science of human-environment interactions, with practical implications for our ability to address the global challenges laid out in the SDGs, is that we generally are not able to measure people in the same way that we measure the environment (Glaser et al., 2012; Kabisch et al., 2015; Dearing et al., 2006; Arias-Maldonado, 2015; Soga and Gaston, 2020). Large-scale ‘big’ data on sustainability are, at present, predominantly focused on environmental variables. These data include, for example, high spatiotemporal resolution satellite imagery, as well as on-the-ground sensors (e.g., high-frequency flow gauges monitoring across watersheds; Hürlimann et al., 2019; Shekhar et al., 2017). These data mean the observation of natural phenomena can be regular, be highly resolved in space and time, cover vast extents (often global), and be representative at many scales. Importantly, this regularised ‘baseline’ measurement of how things are enables natural scientists to identify and speak of ‘anomalies’—in surface temperature, rainfall, etc.—that stand out from the mean and are worthy of examination and explanation.

**Fig. 1:**
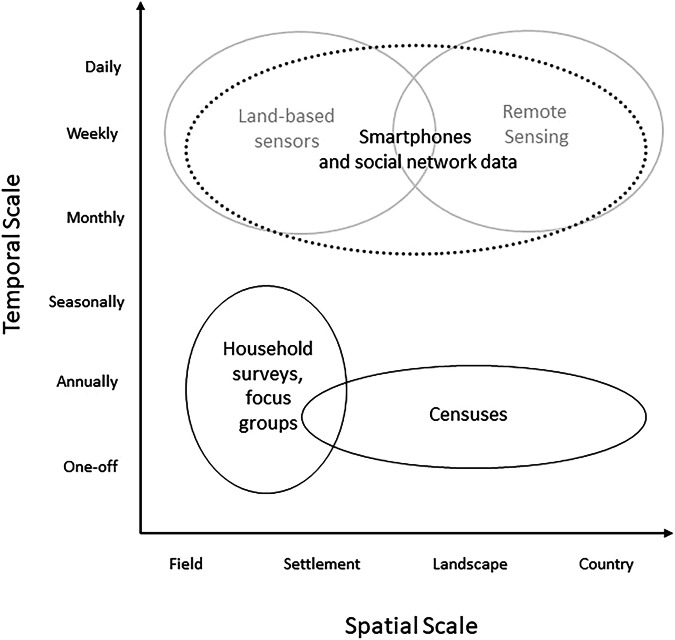
Smartphone surveys (dashes) have the potential to capture socio-economic data at both a high spatial scale as well as at a high frequency. With the rise of smartphone ownership and connectivity across the world, this enables social data (black) typically collected at low temporal scales to match the higher frequency and scale associated with environmental data (grey). Adapted from Willcock et al. (Willcock et al., 2021).

Data collection in the social sciences, in contrast, does not typically allow anomalies to be robustly identified or examined as data on baselines are sparse. Socio-economic data are typically either collected at a larger spatial scale but infrequently (e.g., census data conducted every 5–10 years) or as a snap-shot (Willcock et al., 2021) (e.g., one-off household surveys covering a relatively small geographic extent; Fig. 1). Longitudinal (panel) socio-economic data contain information from the same participant over an extended period of time and can provide valuable insights into changes in behaviour, attitudes or socio-economic characteristics. Such longitudinal data collection is frequently enumerator-led, where trained individuals interact with a participant via a face-to-face interview or phone call (Brück and Regassa, 2022). The expense and logistical challenge of these efforts preclude data collection at the frequency and extent necessary to capture the socio-economic drivers or responses to key sustainability challenges, which may occur on monthly, weekly, or daily timescales across nations/continents. Increases in frequency and/or extent of this data collection require additional person-power, as well as increased travel and subsistence for enumerator-led surveys. As such, the cost and logistic challenges of engaging respondents, coupled to the sheer volume of different things to ask respondents, and variation in respondents’ capacity and willingness to answer, mean that a social data collection campaign may be extensive in geography, broad across subject areas, and frequent in engagement—but typically not more than one of these at any one time. We have simply lacked the resources to engage regularly with large numbers of people when things are not going wrong—when funding is sometimes made available (e.g., during the Covid-19 pandemic; Nguyen et al., 2024).

McCubbins and Schwartz (1984) proposed a framework of monitoring that parallels emergency services. This framework divides socio-economic data collection into two categories. Studies can be reactive and event-driven (termed ‘fire alarms’ within this framework), whereby data collection and studies are initiated in response to a situation thought likely to have caused changes in socio-economic characteristics (McCubbins and Schwartz, 1984). For example, Bakker et al. (2019) used mobile data to analyse the social integration of Syrian refugees in Turkey by evaluating call frequency and duration in reaction to the Syrian civil war (Bakker et al., 2019). However, by lacking a baseline of the levels of social integration before the crisis, the insight gained on the impacts of these changes is limited. For example, fire alarm data campaigns following calamities (e.g., episodes of mass displacement) preclude us from learning what conditions might have led to resilience against these disasters.

By contrast, aligning with natural science data collection, baselines and anomalies could be identified through regular search and observation (termed a ‘police patrol’ by McCubbins and Schwartz (1984)). In conventional models of social data collection, police patrols have been expensive—census campaigns or integrated household surveys—and typically infrequent or of low coverage. However, the frequency of data collection may impact the recall ability of participants (Bell et al., 2019). Consistent, police-patrol engagement with diverse populations could provide novel insights into societal conditions and how these change. Drawing on examples relevant to the SDGs, by contrasting regular baselines under ‘normal’ conditions with those during extreme weather events, insight can be gained into sustainable food production systems and identifying farmers that showed little/no socio-economic change across both periods could help make other farmers more resilient (Target 2.4; UN, 2015). Similarly, insight can be gained to facilitate migration (Target 10.7) and help eliminate trafficking and sexual and other types of exploitation (Target 5.2) by having data on what socio-economic characteristics support resilience to these events by contrasting ‘police patrol’ data from before and after the event (UN, 2015).

The recent global spread of smartphone technology is allowing researchers to reduce the cost barrier of regular engagement in data collection (Bell et al., 2019), making ‘police patrols’ more feasible. Nearly 75% of the global population, aged over 10 years old, now own a mobile phone (though ownership remains higher than internet connectivity, especially in low-income countries; ITU, 2022). This makes large-scale online, short messaging services (sms) and smartphone surveys possible. However, in low-income regions, many still own basic phones rather than smartphones which impacts potential survey formats (Silver and Johnson, 2018).

Researchers are increasingly using autonomous longitudinal approaches, via pre-recorded phone interviews or text message-based surveys, though these approaches favour shorter, simpler questionnaires (Gourlay et al., 2021). Such approaches allow capture of short-term variation and minimal recall losses, with text message based surveys also allowing participants to self-administer the survey in their own time, in their own spaces and without pressure or expectation from an enumerator—providing opportunity for representation that many socio-economic datasets do not offer (i.e., capturing those who are unavailable at the time enumerators visit; Bell et al., 2019). Remotely-led research proved essential during the COVID-19 pandemic, ensuring the safety of both researchers and participants (Bundervoet et al., 2022). Large-scale online surveys share these benefits, also providing the opportunity for longer, more complex questionnaires, though longitudinal research can be challenging, and so high temporal frequencies are difficult to achieve (Bell et al., 2016). Smartphone surveys build on these benefits by also supporting the collection of multiple data types (such as recording sound, visual imagery, or GPS [i.e., routes to a natural resource]) or ‘nudging’ participants to complete the survey (e.g., using automated smartphone notifications to reduce attrition rates). Smartphone surveys can be conducted at high temporal frequencies in locations with patchy or intermittent data connections, at a time convenient for the participant and using free, open-source software, such as Open Data Kit (ODK) (Hartung et al., 2010a).

We believe self-administered smartphone surveys provide an opportunity for a currently underutilised but affordable alternative to traditional enumerator-led surveys to start filling the data gaps required to undertake ‘police patrol’ surveys and help address the SDGs. For self-administered smartphone surveys, research funds previously used for enumerators can, instead, be channelled directly to participants to compensate them for their effort. The proliferation of mobile wallet services—to pay for energy, utilities, or to share money or data—and the expanding demand for data and bandwidth provide the opportunity to reward respondents in locally relevant ways for engaging regularly with data collection campaigns through their mobile devices. For example, following a ‘micropayments for microtasks’ approach (Kittur et al., 2008; Bell et al., 2016) developed a method to collect high-frequency social data by distributing smartphones (that participants could keep at project end) to almost 500 individuals and compensating participation through data and talk time over a 50 week study in Bangladesh. Thus, the impact of these projects can go beyond filling data gaps by providing increased access to technology and information (i.e., via the internet), especially in low-income countries.

**Fig. 2:**
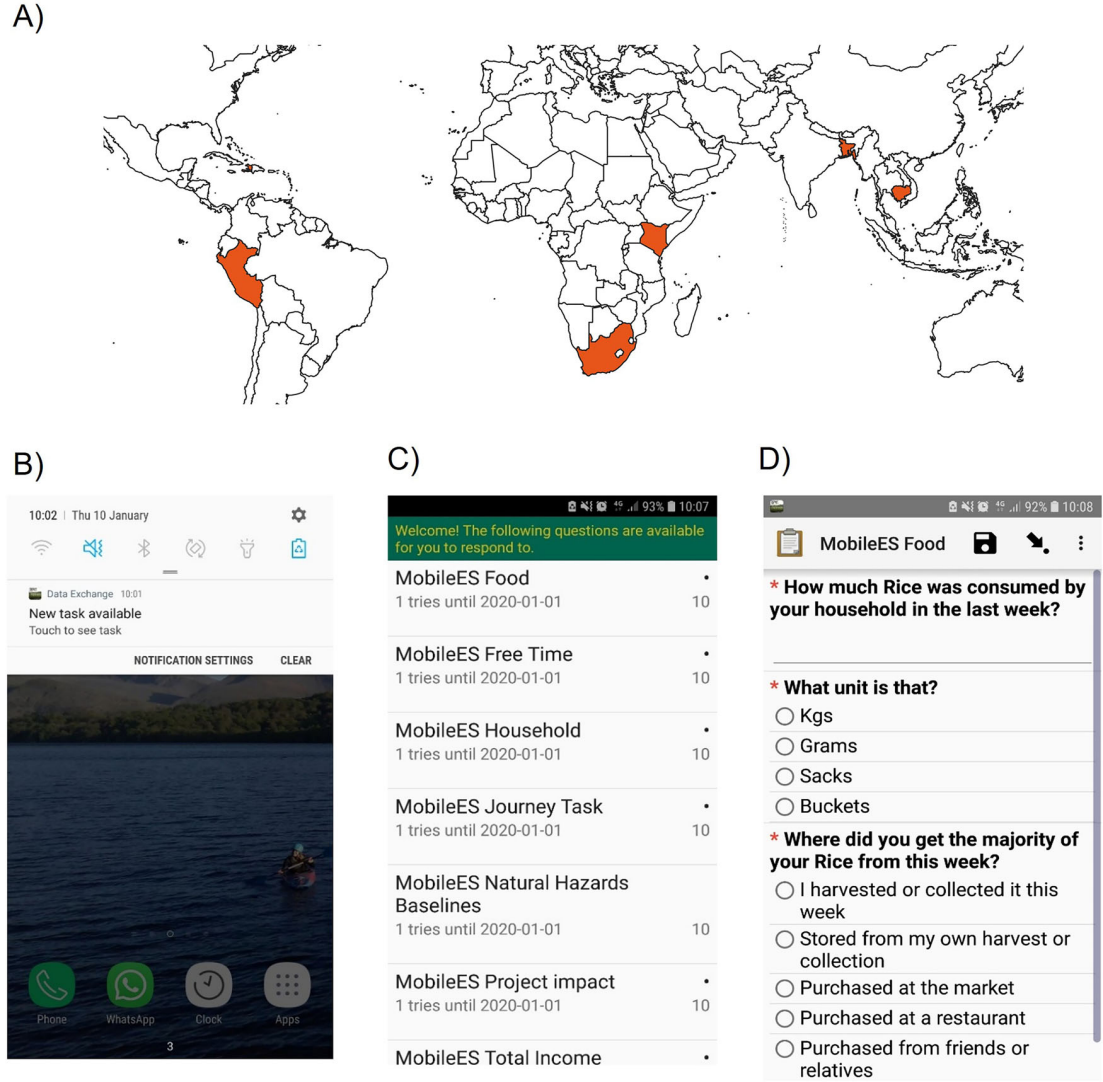
High spatiotemporal, self-administered smartphone surveys (S4) have previously been trialled in six countries between 2015 and 2023 with almost 900 total participants. **A** The location of these six countries. An adapted ODK interface provides: **B** a notification reminding participants that there is a task available; **C** an indication of the points available, the subjects, and the length of the task; and **D** the task itself with the ODK-designed survey, including photo, free text, and multiple-choice options among others.

**Fig. 3:**
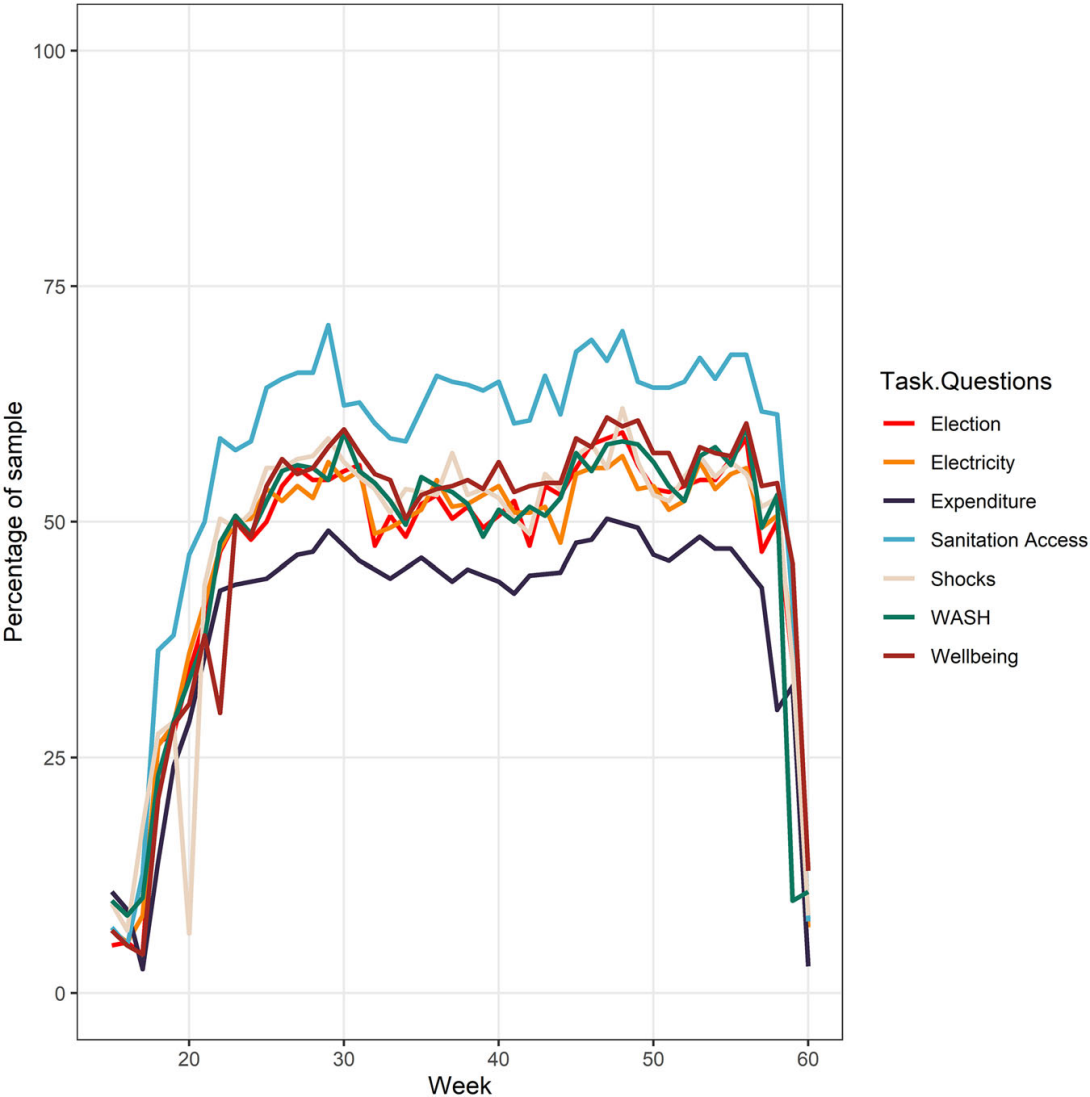
Data from the 2023 studies in Kenya, South Africa, and Peru showing completion rates of surveys by task questions over a one-year period. Topics include water access, sanitation, and health (WASH), as well as shocks and well-being Lewis et al. (2024a; 2024b).

To date, high spatiotemporal, self-administered smartphone surveys (S_4_) have been run in vulnerable communities across multiple countries, including Bangladesh (above), Cambodia, Haiti, South Africa, Peru, and Kenya (Fig. 2; Table 1; https://msds.tools/). Participants were provided with smartphones (or used their own) to regularly complete short daily tasks (3–10 min) in return for small payments in the form of data top-ups (average payment per task was between 0.18USD to 0.25 USD). Topics ranged from basic demographic information, household expenditure, to data on shocks experienced or sanitation access (see SI1–SI5 for questions asked, and Fig. 3 for completion of tasks by topic over a one-year survey).

**Table 1:** A summary of the high spatiotemporal, self-administered smartphone surveys (S_4_) previously run in Bangladesh, Cambodia, Haiti, South Africa, Peru, and Kenya.

	S_4_ Case-study sites					
	Bangladesh (IFPRI, 2018)	Cambodia (Willcock et al., 2022)	Haiti (Lewis et al., 2024a; 2024b)	Kenya (Lewis et al., 2024a;. 2024b)	Peru (Lewis et al., 2024a; 2024b)	South Africa (Lewis et al., 2024a; 2024b)
Study year	2015	2020/21	2022/23	2022/23	2022/23	2022/23
Mobile subscriptions as a percentage of population at time of study (ITU Data Hub, 2023)^a^.	83	130	64 (data from 2021)	120	130	170
Number of participants	480	104	0^b^	102	102	104
Survey Language	Bangla	Khmer	Haitian Creole & French	Swahili & English	Spanish	Xhosa & English
Research length (weeks)	50	30	0	52	52	52
Total compensation budget per participant (including phone costs; USD)	110	120	120	145	120	325^c^
Sex ratio (male to female)	8.53	1.76	^b^	0.68	0.12	0.64
Average age	32.9	33	^b^	29	39	39

^a^Note that this refers to the number of subscriptions to a public mobile-phone service as a proportion of the population of the country at that time, see (ITU Data Hub, 2023) for further statistics. Where the percentage is greater than 100, this implies multiple subscriptions per individual.

^b^Note we have included this as a case study to provide insight on the realities of running an S_4_, including when that results in failure.

^c^In South Africa, the team increased the weekly compensation rates over the duration of the project to maintain retention rates.

This paper seeks to qualitatively answer the following questions. What were the lessons learned when rolling out these research studies? What were the constraints? What opportunities arose? Here, we critically reflect on the realities of using S_4_ and review these six field studies from 2015 to 2023, while we aim to reduce barriers to future researchers taking up this innovative approach. We focus on six key areas: project management, designing the survey environment, software, sampling bias, participant engagement, and data management, as well as discussing the novel insights into human-environmental behaviours. Further case-study specific issues and solutions can be found in the Supporting Information (SI1–SI5).

## Project management and ethics

As with any research collecting data on people, the safety of those individuals and their data is paramount, and inherently, with a new method or technology, new ethical issues emerge for consideration (Brittain et al., 2020). In each of our case studies, team leads in all countries sought ethical approval from relevant bodies and informed consent was sought from all survey participants to take part in the studies. As data collection in S_4_ is accomplished using smartphone technologies, directly to an encrypted server, there are no paper copies of the completed survey instruments that could be misplaced or otherwise violate confidentiality. However, smartphones allow for the collection of some highly identifying data (e.g., precise GPS locations), which must be collected and stored with caution. Access to these data should be exclusive to those where it is absolutely necessary, and it should be anonymised (for example, given a fixed displacement, or data aggregated through distance calculations) before sharing more widely, to reduce any threat of deductive disclosure (Sherman and Fetters, 2007). There was also a risk that non-participants may become envious of the participants’ role in the study and the receipt of a mobile phone handset (if applicable), which can create (potentially violent and/or criminal) conflicts in the community. There was a risk that the handset may be stolen, or their own phone may be declared stolen or broken, so they can receive a new one.

As with other forms of survey data collection, S_4_ requires substantial investment in survey design, code development, and (where required) translation. However, S_4_ has the additional step of server preparation (i.e., encryption, etc) and upload. That said, the total effort required to support S_4_ need not be greater than traditional surveys, as, for example, ODK allows for the development of numerous data input rules, resulting in reduced data cleaning prior to analysis. There are a number of free training resources online, and as with many open-source software, a large community of users willing to help each other (for example, ODK has over two million users across the globe; ODK, 2023). S_4_ may also require additional training at the project level. The case-study projects developed a series of training videos (Lewis et al., 2024) for research and implementation partners to enable them to understand the processes involved in the survey development and roll out (Lewis et al., 2024). In addition, partners developed help/troubleshooting materials such as screenshots and videos, which could be sent via messages (e.g., WhatsApp) directly to participants and team members.

## Designing the S_4_ environment

S_4_ data spanning 30–52 weeks with almost 900 participants across six countries with varying rates of smartphone prevalence have been collected and published (Table 1). Using an adapted interface of ODK (Hartung et al., 2010a), participants have had the opportunity of regularly completing short daily tasks (3–10 min) in return for small payments in the form of data top-ups (0.18USD to 0.25 USD). The microtasks included various topics, from basic demographic information and expenditure to data on economic or environmental shocks experienced, sanitation access, or harvest yields (Fig. 1).

**Fig. 4:**
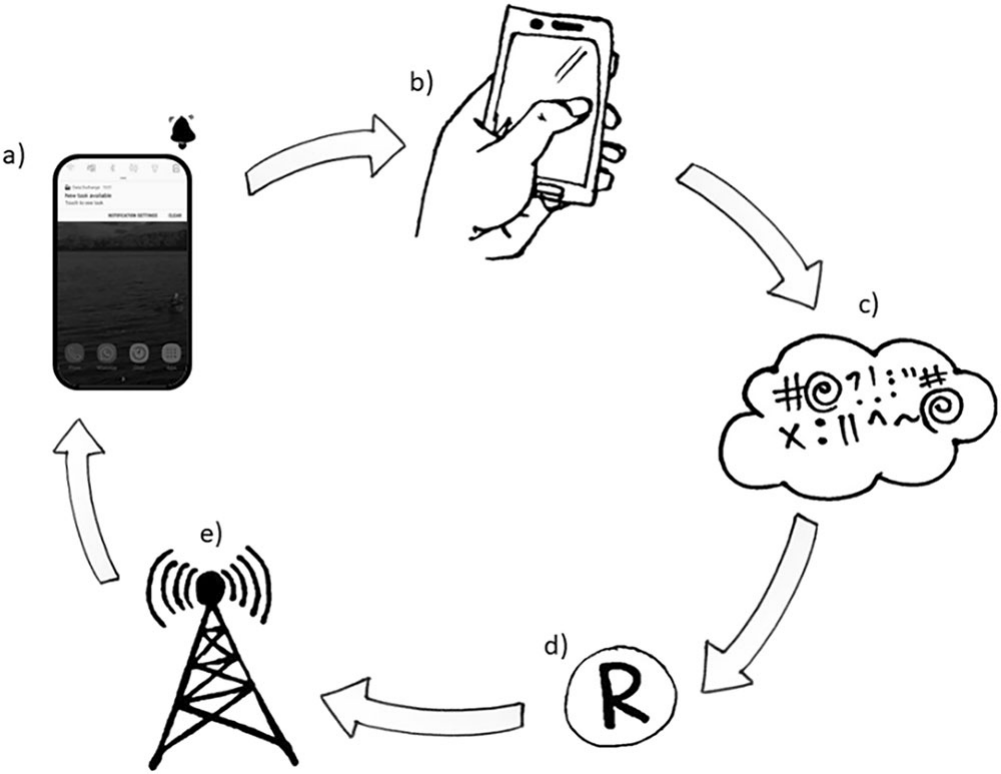
The Data Exchange system. This shows **a** the adapted interface giving notifications and a filtered list of microtasks to complete used in all case studies (Table 1). **b** Participants were taken to the correct task in ODK. **c** Once completed, forms were encrypted & sent to a server when there is a data connection, **d** data were scraped from the server at regular intervals to calculate the ‘top ups’ due to participants using the R package RuODK (Mayer, 2021). **e** Top up values were sent to mobile providers and sent directly to participants’ devices as compensation. Illustrations by A.R.L.

In these case studies, the microtasks were pre-loaded onto a smartphone during a training and consent collection workshop. An adapted ODK interface (Data Exchange) was available to download via the Google Play Store or loaded directly to the phone. The Data Exchange app allowed the notification of a new survey that day or reminded them that a micro task was expiring (Fig. 4a). Participants clicked the notification and were taken to the correct micro task that had already been pre-loaded onto the device. On completion of the task, the data was encrypted and sent (when a required signal is available) to a server (Fig. 4b, c). Data was then pulled from the server, decrypted and unzipped using the R package RuODK (Mayer, 2021; Fig. 4d). Based on the relative difficulty of each micro task, participants were compensated for their effort by adding data or talk time to the phone number linked to that device (Fig. 4e). Participants could use their own phone or be given a project phone. If given a phone, the data top-up budget was adjusted to ensure that the compensation per participant was equitable.

The cost of S_4_ extends beyond phone and data costs. To enable participants to reliably self-administer the surveys, training workshops were required, followed by regular feedback sessions, participant check-ups, and software update workshops—all of which added to the overall cost of the data collection. For example, in Kenya, monthly workshops were held, while in Cambodia, monthly phone calls, ‘check-ins,’ were conducted. In South Africa and Peru, the teams used WhatsApp to communicate with participants. In South Africa, the team set up a WhatsApp group for individuals to raise concerns on an ad hoc basis, alongside one-to-one communication with participants, and the team arranged bi-weekly ‘technical support meetings’ with participants who could not resolve their queries remotely. The team also trained some participants to assist other participants. Such support was a necessary step in ensuring consistent engagement and reliable data, but it added project expense and time costs for the participants.

## Software

Whilst smartphone prevalence has increased (ITU Data Hub, 2023), these statistics may overestimate the levels of smartphone ownership capable of inclusion in S_4_. For example, ODK only functions with an Android operating system. Similarly, even some Android smartphones may not have the capabilities to run ODK (e.g., with poor GPS quality and/or storage capacity). In Peru, it was found that while some phones worked initially, these ceased to function after six months due to outdated systems. In South Africa, in some instances, models of the same brand faced different challenges with running ODK and the Data exchange app. For instance, one model of Samsung worked particularly well with ODK, whereas other models, together with some low-end brands, were unable to run one or both applications. Purchasing higher-end phones added additional cost to the project, and some of them still did not work optimally. Although ODK needs very low storage capacity, many participants’ smartphones have limited or zero storage capacity, and there is fear that ODK can slow down their phones. In South Africa, many participants were receptive to the idea of being issued memory cards to expand their phone memory, although this was not done, as all participants were instead given project smartphones. It was noted that while most of the surveys could run offline, having a good internet connection to initially download the apps and surveys was essential.

Extensive testing of smartphone handsets is required prior to distribution, but it depends on the smartphone handsets in each country. For example, we found that handsets cost more in Peru than in South Africa or Cambodia. In some cases, Android updates forced a reboot and required a reinstallation of the apps, and while this did not lose data, this required input from researchers and frustrated participants. To combat this, we found that remote access software such as AnyDesk (AnyDesk, 2023) worked well (e.g., in Kenya); however, this software was not used in South Africa because of higher data costs. There were also regular in-person ‘technical support meetings’, calls, or materials sent by WhatsApp with step-by-step processes. However, to make support clinics work, participant engagement is needed so that issues can be properly diagnosed and addressed.

During the collection of data, our case studies show that multiple communication channels were often required to ensure a good participant experience. For instance, while S_4_ can be conducted predominantly remotely, frequent in-person visits were included in most cases. In Kenya these were mainly in the first three months, while in South Africa they were done throughout the survey period. This meant increasing the number of research assistants and their hours of work than had been planned initially. Country teams shared experiences each month to learn and add new strategies that might work in each context.

The S_4_ examples used here also faced a series of software-based setbacks. The original Data Exchange app, developed in 2015 for the Bangladesh survey, was rendered obsolete as a result of Android updates (Android Developers, 2023) by the time the project in Cambodia was rolled out in 2019. New Google PlayStore updates (GooglePlayStore, 2023) also meant that by 2021, the rules by which Data Exchange and ODK were able to ‘communicate’ changed, and a further overhaul of the app was required. Such software development is likely to be a continuous process. However, this also potentially unlocks exciting new features. For example, ODK-X allows fully customisable user interfaces (obviating the need for a layer like Data Exchange), as well as bi-directional synchronising between device and a cloud table (allowing easy reference to past responses and the dynamic updating of survey tasks; ODK-X, 2018).

In each case-study country, S_4_ was provided in multiple languages, with a default language set using the app settings (for example, in Kenya, someone could participate in the survey in Swahili, but intermittently switch over to English if required or vice versa). Enabling multiple languages as a default increased the workload in terms of data management and uploads, with few economies of scale (i.e., each additional language requires the same workload as the last). This benefits bilingual participants, but also enables easy replication of surveys once the translations are complete.

## Representativity and inclusion

While smartphone access and adoption vary within and across the case-study countries (Silver and Johnson, 2018), it is reasonable to expect those participating in S_4_ samples to skew toward those with greater technical literacy and written language fluency. However, barriers to engagement via mobile device are falling over time with the growth of smartphone use in everyday life across urban through rural spaces (ITU Data Hub, 2023). Moreover, the S_4_ approach can also be more inclusive of busy people (who might not have time to take away from work or other responsibilities to participate in a conventional survey) or those who may be more traditionally marginalised in having their voices heard (Grossman et al., 2014). The risks of self-administered questioning include pinpointing which member of the household completes the survey. To reduce this risk the case studies included hints and notes during each task to ensure continuity of participant (Lewis et al., 2024a, 2024b). Conversely, the S_4_ approach may additionally give a perception of anonymity, allowing for the discussion of sensitive issues which participants may not wish to discuss face to face with a stranger (e.g. sanitation; Schonlau et al., 2002). S_4_ can be made more representative by participant-driven sampling (reaching out through networks and leveraging trusting relationships to reach people who might not otherwise engage in a conventional survey; Bell et al., 2016).

An emergent challenge with the novel, non-enumerated smartphone approach is that of linking observations from this method with those collected in other efforts. Responses to any one survey task are understood to be a co-production of the respondent with the larger survey instrument and the context (Fielding and Fielding, 1986)—including the enumerator (Di Maio and Fiala, 2020) (or lack of), along with all other observed and unobserved aspects of the study frame. Thinking carefully about how to make comparisons across, or construct time series linking such different survey modalities—where respondents will have responded with different motivations to round numbers, think expansively, or misrepresent, for example—is a critical challenge for enmeshing smartphone-based survey research as a modern data collection paradigm.

We acknowledge that the S_4_ approach can present additional challenges for individuals living in very poor data connection environments. This was seen in the South African case study, for instance, where there was a high incidence of power and network disruptions. Many participants also migrated seasonally to rural areas with poorer network connections (Biswas and Mallick, 2021). The method is robust to low and intermittent connectivity, such that completed surveys can be sent at the point when an individual does hit a data connection, but the experience of S_4_ participation is smoother with a reliable internet connection.

There is no ‘one size fits all approach’ to the question of whether smartphones should be given to participants or whether they should, or would choose to, use their own. In some of these case studies, a few mobile phones were handed out, with many participants preferring to use their current phone. If a phone was not given to a participant, they would instead receive greater top-ups of data or talk time to ensure equity across participants. In Peru, only 10 of 102 participants required a project phone, which were handed out discreetly. However, in South Africa, all 104 participants received a project phone, although there can be substantial challenges associated with this. The academic partners managing the project in South Africa were acutely aware that it would be irresponsible to distribute 100 smartphones in a small geographic area, where residents faced high levels of economic precarity. This could cause conflict between participants and non-participants. Initially, the facilitators did not explicitly state to participants that they could have phones given to them, and thus only those with smartphones, with capabilities to run ODK and Data Exchange, began completing the survey. The facilitators shortlisted those who did not own smartphones and discreetly provided them with the ones purchased under the project. Over several months, however, it became clear that (a) some of the low-end phones that participants owned did not work smoothly; (b) news of phones being given to some participants spread and there was resentment felt by those who were using their own smartphones and had not been given the option to use a project phone (which in turn led to drop outs); and (c) retention rates decreased for those using their own phones as the micropayments were no longer seen as a sufficient incentive, compared to tangible smartphones despite having the same overall monetary value per participant. To maintain the survey, the facilitators individually met with participants discreetly and, ultimately, gave every participant a phone. In Kenya, 20 participants used their own smartphones the remaining 80 took project phones.

The Covid-19 pandemic increased not only access to better-quality phone ownership so families can engage with schoolwork but also internet access (Aguilera-Hermida et al., 2021; Kadada and Tshabalala, 2020). Despite the rise in connectivity across the world, there is a significant gender digital gap with fewer women accessing technology (Mariscal et al., 2019). In most case studies, there was a skew towards female participation in the research projects (Table 1). There was a skew towards younger participants; very elderly participants often did not want to participate. Though not unique to S_4_, in some contexts, it is not feasible to roll out the surveys (Table 1). In Haiti, for example, the environmental and political situation was substantial (Keen et al., 2020). There were also extreme import costs on devices, as well as significant energy instability.

## Participant engagement

Gaining the trust of participants was something that researchers in Peru needed to overcome at the beginning, with a suspicion of cold callers. The recruitment strategy had to work closely with local leaders who reinforced the invitations to the training workshops, and the research team added more female personnel to the team to give a sense of trustworthiness. As above, additional workshops throughout the project were required to maintain engagement, and attrition rates across the case studies varied (see SI1–SI5). In South Africa, mistrust between researchers and participants developed when project smartphones were reported lost amidst rumours that some had sold them. Community leaders who were already participants intervened and/or were engaged by researchers to urge participants to keep their project smartphones safe. Regular meetings were held with participants to rebuild trust and, overall, many participants remained engaged (see case-study specific examples in SI1–SI5).

Regular near-real-time data analysis and concomitant feedback would have benefitted researchers in testing the app ‘engagement’ and allowed checks of the data; however, this would require additional data analyst time or automation. Additionally, in South Africa and Peru, participants felt that answering the same questions each week was repetitive. Confusion can be caused by inadequate explanation of the longitudinal format of the survey (e.g., in South Africa, some participants thought there was an error due to the repetition of the questions). While this may be true for all longitudinal surveys, new software developments can enable researchers to focus more on user experience and ask dynamic questions.

Furthermore, cultural, contextual, and scale-related differences may impact survey responses (Balsa-Barreiro et al., 2022). The high spatial and temporal resolution data across large extents that are possible under S_4_ enable this to be studied, with the ‘police patrol’ nature of the methodology enabling for these differences to be controlled for when studying an event by contrasting the baseline context via the socio-economic data collected after the anomaly.

## Data management

In all case studies, regular data scraping and points calculations were both required to give consistent top-ups, but also to flag participants who had not submitted data for several weeks. The flagging of such participants also aided the process of identifying and resolving challenges, technical and otherwise, that would have prevented them from completing the survey. However, this required a weekly or bi-weekly commitment from researchers both centrally and in each of the field sites. Novel R code (Mayer, 2021) was developed to enable ease of data pulling from a central server, vastly reducing download times (Lewis et al., 2024a, 2024b). In addition to top-ups, there were further data management requirements when collecting that volume of information, and the capacity gaps in analysing socio-environmental data at these scales now need to be addressed.

## Novel insights into human-environment behaviour

We believe that there are good reasons to shoulder the burdens in S_4_ data collection that we have outlined above. Asking participants to complete short tasks, regularly and on their own time, can bring patterns of engagement and recall that are typically not possible in conventional surveys. Additionally, the high-frequency insights into participant experience that smartphone-based engagement provides facilitate time series analysis at the level of shocks and decisions. Further, it allows us to move from point estimates of highly variable aspects (e.g., consumption, spending, and access) to describing the moments of their within-subject distribution over time (e.g., mean, variance, skew). For example, Adams et al. (2016) clustered rural participants in Bangladesh by the ‘shape’ of their reported well-being over time, finding that the role of shocks in shaping well-being was different across clusters—an insight that would have been missed in a conventional survey. Where surveys provide measures of related variables at high frequency, this same approach can extract their covariance as a key outcome variable that may be predicted by other characteristics of the participant (e.g., the degree to which variation in food consumption shapes well-being).

**Fig. 5:**
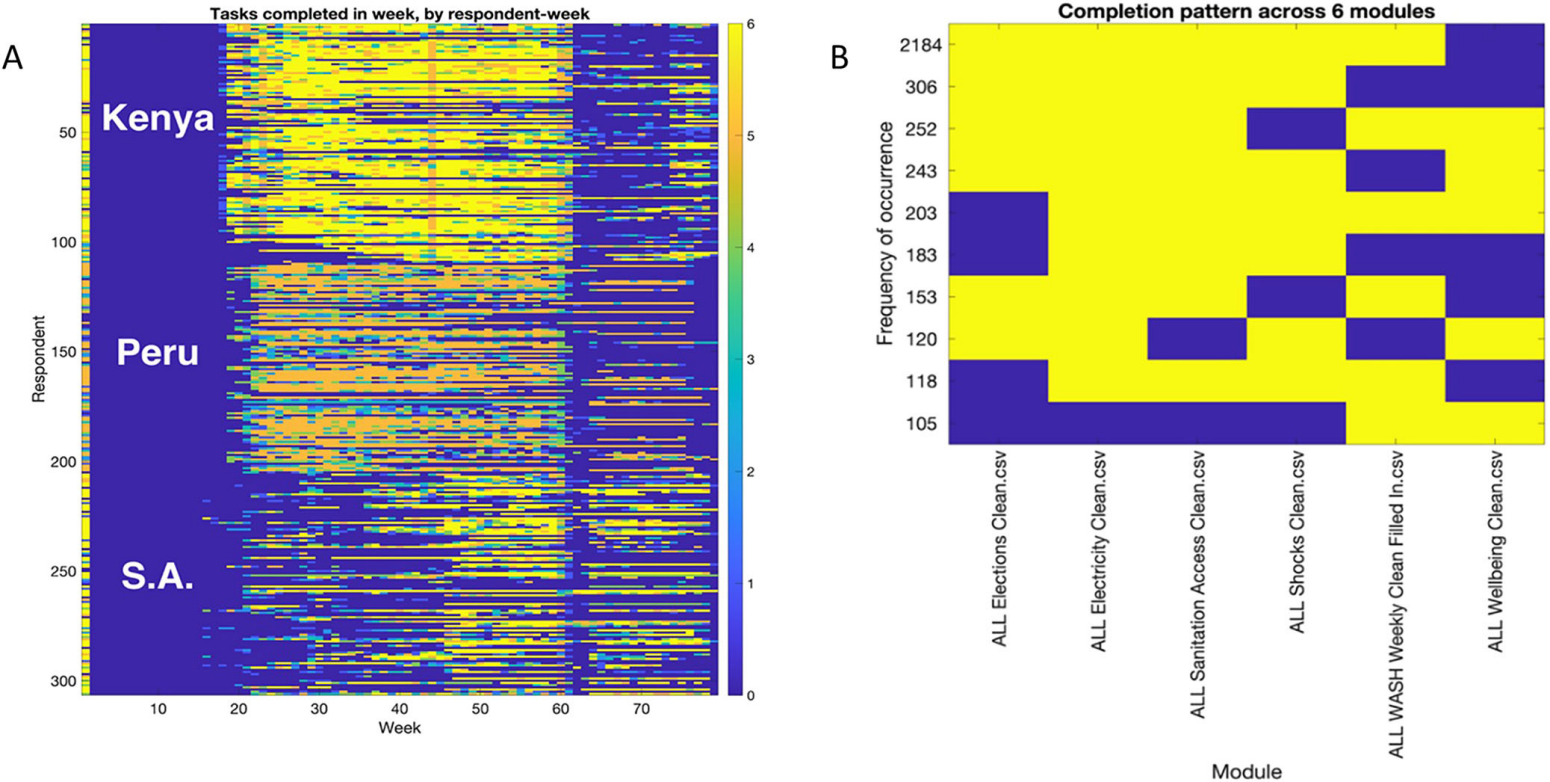
An example of high-dimensional participation analysis on S4 from 2023 studies in Kenya, South Africa, and Peru (Lewis et al. (2024a)). **A** Multi-country dataset showing the number of tasks completed by respondent, by week; **B** identification of the highest-frequency non-engagement patterns in the dataset shown in (**A**).

In addition to these high-resolution time series analyses and ‘shape’ analyses, the high frequency, high-dimensional picture within and across participants provides other novel opportunities. For example, it gives us the capacity to identify specific events or shocks within the study period as units of analysis, or to identify broader patterns across the sample that might have been invisible in a conventional survey (e.g., patterns of response and non-response to specific question types or over specific survey periods; Fig. 5). These novel lenses into human response are immensely valuable in understanding the kinds of adaptation to shocks that strongly shape how people, communities, and societies will be able to respond to interventions aimed at advancing toward the SDGs.

## Discussion

If we want to achieve the SDGs by 2030, we must rapidly and robustly fill the data gaps in human-environmental interactions (Glaser et al., 2012; Kabisch et al., 2015; Dearing et al., 2006; Arias-Maldonado, 2015; Soga and Gaston, 2020). Where environmental data collection has leapt ahead in its ability to collect high spatial and temporal resolution data across large extents, socio-economic data must catch up. The expansion of smartphone ownership and data connectivity gives us an opportunity to address some of the most pressing challenges of our time, such as climate change and inequality. Already, S_4_ has shown that people can recall their past activities reasonably well, but not their past consumption or their experience of shocks (e.g., illnesses and missed school days (Bell et al., 2019). S_4_ has also shown that the ‘shape’ of how people report their well-being predicts how they experience shocks (Adams et al., 2016).

Rolling out an S_4_ is certainly not without its challenges. However, most of these challenges are not insurmountable. Software development, troubleshooting forums, and an increased willingness to share in failures as well as successes will reduce these barriers. Channelling resources typically used for high-cost field studies also increases the direct impact of research funding on participants. This method, therefore, enhances ethical research by appropriately valuing both participant data and time. However, sensitivity is required when introducing technology into low-income communities. In settings where there are high levels of economic precarity alongside high-density living, a smartphone or regular payments for research input can cause conflict between neighbours. Time and effort must be put into the sampling method, and trust must be built in the area in which researchers plan to work. Local leaders also play an important role, and should have knowledge of the project, as they will often have to mediate any conflicts that arise. Smartphone technology and the apps used for data collection can be alienating and frustrating for participants when they do not work, data is low, or connectivity fails. However, as software improves, these challenges are likely to be overcome.

The use of smartphone technologies to collect data for social research also has some limitations. A key limiting factor is people’s willingness to participate in the smartphone survey. Literature shows that two main aspects have an impact on people’s willingness to participate in these types of surveys, i.e., respondent characteristics and study characteristics (Wenz and Keusch, 2023). In terms of study characteristics, one of the key factors is the duration of the study. Participation in smartphone surveys can be low, and participants tend to prefer short studies run by universities rather than sponsored by companies or agencies (Wenz and Keusch, 2023). The examples discussed here had different attrition rates (e.g., Kenya: 73%, Peru: 54–55%, South Africa: 29–55%) and different incentive mechanisms (Lewis et al., 2024a; 2024b), which may have also had an impact in survey participation (Wenz and Keusch, 2023). Therefore, keeping participants engaged, especially when they need to respond to high-frequency tasks, remains a challenge, and it is vital to ensure data quality.

Such a paradigm shift in data development towards S_4_ also brings risks and challenges. Differences in engagement and response present challenges in linking smartphone-based responses to conventionally derived responses. Variation across respondents’ interests and capacity may lead to variation in data quality, as well as gaps in engagement, which is difficult to control for. As with other survey methods, there is the possibility of misreporting by participants (either deliberately or accidentally) via the provision of biased responses (Bach et al., 2020). However, we note that the frequency of data collection made feasible by S_4_ may increase the accuracy of reporting by minimising the recall time required (Bell et al., 2019). That said, that does not mean that the data quality of the data collected from the participants will not be challenging. Thus, the dataset collected had to undergo extensive data cleaning to remove duplicate answers, out-of-date responses, and to ensure that responses were allocated to the correct weeks. Full details of the cleaning protocol are covered by Lewis et al. (2024a, 2024b).

Given that the number of S_4_ is rapidly increasing, there is future scope for a meta-analysis from smartphone survey studies across multiple countries to provide quantitative insights into this methodology. Potential future investigations could answer questions such as: What are participation (who joins) and retention (who stays) rates in S_4_? Are these rates representative? i.e., How do retention rates vary with socio-economic variables? If retention rates are lower for certain groups, a representative survey may start with good representation but can become increasingly skewed with time. Even when retained, the frequency of engagement of participants of self-administered surveys may vary—how does the frequency of engagement vary with socio-economic variables? How do response rates vary per question type? Are some types of questions (e.g., quantitative vs qualitative, photo vs GPS tracking, etc.) considered easier for respondents (e.g., reduced response time), leading to reduced attrition? Can we identify underlying dimensions of ‘participation’ that show connections across thematic areas or patterns over time? (Fig. 5); and finally, what is the optimal survey period and resolution required to capture information on anomalies?

## Conclusion

The lack of understanding of baseline socio-economic conditions is a key limitation to traditional crisis-driven data collection methods, such as surveys conducted in the aftermath of natural disasters. However, the widespread adoption of smartphone technology (ITU Data Hub, 2023) has significantly reduced barriers to social data collection at high spatiotemporal resolutions and across large extents. This proliferation of smartphones makes S_4_ feasible, enabling us to have consistent engagement with participants of a longer time period, capturing novel insights to socio-environmental systems. Despite this progress, disparities in technology access persist (Mariscal et al., 2019), particularly in low-income regions where ownership of basic phones outweighs that of smartphones, impacting the potential efficacy of survey formats. While technological advancements have opened new avenues for data collection, efforts must continue to bridge the digital divide to ensure equitable access to information and insights across diverse populations.

## Supplementary information

Lews_SI_final

## Data Availability

The data that support the findings of this study are openly available in multiple repositories Data Verse at 10.7910/DVN/HBQQVE and Reshare at 10.5255/UKDA-SN-854681 and 10.5255/UKDA-SN-857073.
