# Supplementary material for: Realities of using self-administered smartphone surveys to solve sustainability challenges
Source: Humanit Soc Sci Commun. 2025 Jul 19;12(1):1134. doi: 10.1057/s41599-025-05305-w (PMC12276084; doi:10.1057/s41599-025-05305-w)
Supplement: Supplementary file 1 — Lews_SI_final [file 41599_2025_5305_MOESM1_ESM.docx]

## Supplementary Information

SI1. Peru Case study details

In implementing S_4_ project during 2022-2023 in Pamplona Alta Lima, Peru, the team encountered various challenges and devised innovative strategies to address them.

**Security risks mitigation:** Security risks were acknowledged, but a proactive approach, such as calling individuals instead of door knocking to avoid perception of scamming, was adopted. Trust was established through transparent communication.

**Community engagement and expectations:** Concerns about attendance were not realized as workshops successfully involved users and non-users of CBS toilets. Expectations were managed appropriately to avoid disappointment. It was acknowledging the presence of numerous NGOs in Pamplona, collaboration and coordination efforts were crucial to ensure the effectiveness of the CBS data collection project.

**Incentive adjustment:** Initially, the incentive structure faced challenges, with some experiencing reimbursement delays. The team adapted by increasing the incentive amount to $5 to $7 and improving communication for timely top-ups. Despite challenges, the retention rate was commendable. Participants found the incentive meaningful, particularly given the local political situation, and expressed satisfaction with the survey process.

**Technological solutions:** To facilitate communication and data collection, approximately 10 phones were purchased. However, some technical issues remained unresolved, presenting a challenge in the implementation process certain types of phones were not compatible with the apps.

**Continuous communication:** Regular WhatsApp communication between participants and the project team was established, fostering a sense of community and enhancing engagement.

**Lessons Learned:**

1. **Adaptability:** Flexibility in adjusting incentives based on local contexts and economic conditions is essential for project success.
2. **Community trust:** Proactive measures, such as personalized invitations and transparent communication, contribute significantly to gaining and maintaining community trust.
3. **Technological challenges:** Anticipating and addressing potential technical issues is crucial for the seamless implementation of technology-dependent projects.

**Conclusion:** The lessons learned from the data collection in Peru emphasize the importance of adaptability, community engagement, technological preparedness, and collaborative strategies for the successful implementation of sanitation initiatives in diverse socio-political contexts.

SI2. Kenya case study details

The implementation of an S_4_ project in Kenya highlighted the importance of adaptability and strategic planning.

**Household Recruitment and Training:** Initial household recruitment demonstrated success with good representation. However, a significant number dropped off post-recruitment due to technical issues. The team implemented a retraining program to address challenges and enhance participant engagement. During the initial workshop, security risks arose, necessitating police intervention to manage the situation and control access. This incident emphasized the need for careful planning and security measures during community engagement.

**Technical Support and Expertise:** Technical challenges were prevalent, prompting the team to enlist the support of a researcher with expertise in technical areas. Collaborative efforts, including the use of AnyDesk software^28^, proved essential in resolving issues and improving the overall efficiency of the project.

**Engagement and incentives:** To address technical issues and maintain participant engagement, two team members conducted monthly visits to the settlement. This not only facilitated payments but also allowed for real-time troubleshooting and support. The incentive structure, utilizing mobile money transfers and 1 GB of data, proved effective. Participants, motivated by competition with friends and neighbours, considered it a consistent income source. The competition aspect contributed to a sense of achievement and motivation.

**Impact on Participants:** Beyond financial incentives, participants reported increased awareness and learning through the project. The initiative contributed to rent payments for some, and participants expressed satisfaction, noting that their “eyes were opened to new perspectives”.

**Challenges and Recommendations:**

1. **Technical Support:** Future projects should prioritize robust technical support, both remotely and in the field, to swiftly address issues and retain participant involvement.
2. **Infrastructure Challenges:** Power cuts and prolonged periods without electricity were identified as challenges. Strategies for addressing power-related issues are crucial for sustaining project effectiveness.
3. **Community Expectations:** Managing expectations, addressing boredom concerns, and fostering continued engagement are crucial elements for the success of similar projects.

**Conclusion:** The lessons learned from the S_4_ project in Kenya underscore the importance of technical expertise, robust security measures, and thoughtful incentive structures. Balancing competitive elements with community impact can lead to sustained engagement and positive outcomes.

SI3. South Africa case study details

The pilot phase of the S_4_ project in South Africa was deceptively successful while the roll out of the wider scale data collection provided valuable insights, revealing significant challenges and costs associated with S_4_.

**Deceptive Success of the Pilot:** The pilot phase initially appeared successful, but its deceptive nature stemmed from the selective inclusion of younger participants with smartphones, creating a skewed representation. Choosing participants with existing smartphones for the pilot posed challenges as during the roll out of the final project, some devices were non-functional. The majority of participants were relatively young in the pilot, and the one-day intensive session had strong Wi-Fi connections, which would not be true during the wider roll out phase.

**Inclusion of Community-Specific Challenges:** In the South African case, the team recognized a community-specific challenge in BM informal settlement, where a significant portion of residents did not own smartphones. Moreover, among those with smartphones, many possessed low-end devices lacking the capabilities required to run smartphone surveys like ODK (Open Data Kit). This presented an additional layer of complexity, necessitating increased in-person support to sustain project operations. Addressing such community-specific challenges is paramount for future projects, emphasizing the need for tailored approaches and resources in diverse socioeconomic settings.

**Technical support and retention strategies:** Recognizing the need for ongoing technical support, the project team implemented twice-weekly workshops to address participants' technical queries and challenges. The team in South Africa led to the decision not to inform participants that they could keep the phones, aiming to discourage dropouts. However, constant resetting of phones for fieldwork created a culture of resetting, which affected the projects ability to keep track of participants scores, thus increasing frustration and drop outs. The cost of data in South Africa is significantly higher than in other countries. The initial budget was increased in this case study as too many participants were dropping out as the initial incentive was not enough.

Regular phone calls played a crucial role in retaining participants, emphasizing the importance of consistent communication. The project team identified areas for improvement in training, suggesting the need for a more extended training period or a reduced staff-to-participant ratio.

**Phone Ownership and compensation:** Issues of jealousy and interpersonal dynamics emerged as participants were aware of who owned phones. Future projects may reconsider the decision to buy phones or explore options where everyone has equal access to devices.

**Lessons Learned and Recommendations:**

1. **Comprehensive Training:** Invest in more thorough and extended training sessions to equip participants with the necessary skills, reducing reliance on frequent technical support.
2. **Equitable Access to Phones:** Consider alternatives to phone ownership, such as providing devices to all participants (or to non at all), to mitigate interpersonal issues and jealousy within the community.
3. **Improved App Development:** Prioritize the completion and testing of the app before implementation to ensure optimal functionality and user experience. Thorough piloting to highlight any issues with the app and phone capabilities in the field.
4. **Transparent Communication:** Despite high data costs, transparent communication about phone ownership can build trust and mitigate unintended consequences.

**Conclusion:** The South African S_4_ experience highlighted the importance of transparent communication, equitable access to resources, and ongoing technical support for successful smartphone surveys.

SI4. Cambodia case study details

The S_4_ project in Cambodia revealed both successes and challenges, highlighting the importance of addressing hardware issues, enhancing smartphone training, and adapting strategies to local contexts amidst external factors like the COVID-19 pandemic. During this project, the team ran a parallel phone call project to assess the benefits of S_4_ against a traditional weekly phone call survey.

**Project Success and Reception:** The S_4_ pilot project in Cambodia was successful, with widespread community engagement and enthusiasm. However, the phone call project highlighted difficulties in engaging farmers who frequently left their phones at home during working hours.

**Hardware issues:** Hardware issues affected the project, including charging phones in rural areas using car batteries.

**Participation and Incentives:** Incentives, such as providing smartphones, proved effective in motivating participation, especially among rural populations. However, limited smartphone knowledge among selected farmers underscored the need for comprehensive training.

**Impact of External Factors:** The COVID-19 pandemic posed significant challenges, including restrictions and competing livelihood priorities affecting project participation.

**Challenges & Recommendations:**

1. Overcoming the obstacle of farmers leaving phones at home during working hours.
2. Addressing hardware issues related to charging phones in rural areas, for example providing solar chargers.
3. Provide larger phones with bigger font sizes to accommodate users with limited smartphone knowledge and improved font size.
4. Implement more extensive smartphone training sessions, extending beyond a single-day workshop.

**Conclusion**

Overall, the experiences from the S_4_ project in Cambodia underscore the significance of flexibility and adaptability in project implementation, emphasizing the need for continuous evaluation and refinement of strategies to effectively address the evolving needs and challenges encountered in S_4_.

SI5. Bangladesh case study details

The S_4_ project aimed to utilize smartphones for agricultural data collection and dissemination in Bangladesh. Throughout the implementation, various lessons were learned, shaping the project's trajectory and outcomes.

**Smartphone Familiarization:** Training farmers in smartphone usage was crucial, especially considering initial ownership of non-smartphones; involving younger family members aided adoption.

**Addressing Phone Resetting Culture:** Despite training, the prevalent practice of factory resetting phones posed challenges in data retention and task continuity, requiring ongoing mitigation efforts.

**Local Support Network:** Engaging local contacts for troubleshooting and task re-downloading proved essential in overcoming technical hurdles and maintaining project momentum. In total six field staff were recruited to the project.

**Data Utilization Challenges:** Constraints such as the need to consume 100MB of data within two days and task frequency exceeding seven days highlighted the importance of optimizing data usage strategies. In the end points were calculated daily and uploaded to the mobile providers to ensure enough data on participants phones.

**Storage Management Solutions**: Implementing periodic removal of old surveys from the server and uploading new ones helped manage limited phone storage capacity and optimize data management efficiency.

**Conclusion**

The project's journey in Bangladesh underscores the necessity of tailored technological interventions, community engagement, and adaptive strategies to navigate infrastructure and behavioural challenges. These insights are crucial for future initiatives seeking to leverage technology for agricultural development in similar contexts.

SI6. Example Consent Form

Thank you for your interest in the project. This document covers an outline of the research being conducted, information you need prior to participating in the project and contact details should you have any queries.

**Project summary:**

To protect human health and the environment, sanitation systems must separate people from their excreta and treat it. Sewers and wastewater treatment plants can assist in providing safe sanitation, but they are expensive and challenging to build, particularly in dense urban areas or where people do not own the land that they live on.

This research will focus on an emerging off-grid sanitation option in the form of container-based sanitation (CBS) across four city contexts where the provision and regulation of CBS is done by different organisations. In Cap Haitien (Haiti), CBS is provided by an NGO, in Lima (Peru) it is provided by a private company, in Cape Town (South Africa) it is provided by the municipality and an informal settlement in Nairobi (Kenya) it is provided by a private company working with an NGO.

The primary data collection will be through surveys. To enable the collection of a large volume of data over a longitudinal period of time we will use mobile technology to conduct a series of repeating micro-surveys over the entirety of the research period. In each location surveys will be conducted with 50 CBS users, and 50 users of other off-grid sanitation options to provide a control to look at the wellbeing differences and impacts of CBS. The survey will last 12 months, and is designed to address the following questions:

1. To what extent do institutional, legal and regulatory contexts enable the sustainability of CBS business models?

2. Are the designs of CBS services inclusive or do they increase disparities within and between vulnerable groups and individuals?

3. How do off-grid services like CBS impact the mental well-being of users?

4. How does CBS link with other urban services, e.g. water, food and solid waste, and the interactions between them?

5. How might the CBS model be applied to improve other services to cities’ most marginalised populations?

**Project partners:**

This research is being funded by the European Social Research Council (ESRC) and involves researchers in the UK, USA, Haiti, Kenya, Peru and South Africa. The project is led by Cranfield University (UK), but the survey itself is being led by Dr. Simon Wilcock at Bangor University (UK) and can be contacted at [s.willcock@bangor.ac.uk](mailto:s.willcock@bangor.ac.uk). Locally you can call the project manager in INSERT RESPONDENT COUNTRY on 07******** if you have any queries or problems.

**Compensation:**

The project will include multiple short tasks (1-10min in length) each week. Tasks will be on a regular basis from a weekly to quarterly timescale (with a maximum of 10 short tasks per week). Each task will have a set number of points depending on the difficulty of the task and the time required to complete it. These points build up and each week you will be given data and talk time corresponding to the number of tasks you have completed that week. In total, if you complete all tasks, you can expect to receive compensation of INSERT USD (0.05-0.1 USD per task, in form of data, sms, and/or credit toward device ownership). If you do not already own a smartphone, one will be provided for you, and some of your points will go towards earning ownership of the smartphone. If you already own a smartphone, our app can be installed on your phone and your points will all go towards data/sms credit.

It is important to remember that the smartphone credits you receive are more than enough to cover participation in the survey and, as such, you will not to purchase air time to be able to respond to our survey (but you may wish to purchase additional air time for other uses). You **will not** be penalised for failing to complete a task. There is a small risk that other members of the community may become envious of the your role in the study. If the smartphone we provide is broken/lost/stolen, you will not have to reimburse us for the cost of the phone, even if you were yet to earn full ownership; however, this may mean you are no longer able to participate in the project, unless a replacement phone can be found. Other than this, and the time you are committing, we do not perceive any significant risks to social, emotional, psychological, or physical status/wellbeing.

Finally, we also hope that your participation will be of benefit to the wider community. We are working in close collaboration with local sanitation organizations who are keen to use the information gained via this project to enhance their ability to address local sanitation problems. We will share anonymized project findings with them and, as such, we hope that this will lead to improved sanitation processes for the local community in the future.

**Consent:**

The consent you provide here will cover all tasks you are asked to participate in throughout the 12 month period.

1. We will keep all the information you give us confidential. While the project is ongoing (from now until 31/3/23), we will not share your personal details or personal views with anyone else.
2. Some of the information you give us may be published, but your real name will not be used in relation to any of the information you have provided us, unless you tell us clearly that you want us to use your real name.
3. You should know that even though we will avoid including identifying information in any publication, there is still a possibility that people will recognise you by the things you say. If at any time you feel concerned about what you are saying being disclosed, please feel free to stop and talk to us about it.
4. Any photographs you are asked to take within a task may be used to share with stakeholders. Remember not to take a photo within a task unless you are happy for it to be used for this purpose.
5. You can stop participating in this task, at any time, without giving us any reason. To do so, simply stop responding to the surveys. If you do this, you may receive a phone call from us, just to check you are not having any difficulties with the survey. If you inform us at this time that you wish to stop participating in this task, you will not hear from us again. However, you may be asked to return the smartphone.
6. As well as stopping to participate, at any point during the 12-month survey you can also request for any/all data your provided to be deleted. This can be done by contacting the project manager on 07********, or by raising this if/when you are called by us.
7. Do you have any further questions? Please raise any questions to the workshop organiser
8. Do you agree to participate in this project? YES / NO

(please delete as appropriate)

1. Shortly after the project has completed (approximately 31/3/23), with your agreement, the data collected will be made available online, following rules and guidelines in the United Kingdom designed to ensure your safety and privacy. This data will be de-identified (i.e., names will be removed, but precise locations will be retained). The depositor will be Dr Simon Willcock, Bangor University. Here, we seek your permission for this:

*“I give permission for the de-identified survey data that I provide to be deposited in the UK Data Archive repository so it can be used for future research and learning. The data will only be made available with the depositor’s approval”.*

Do you agree with this statement? YES / NO

(please delete as appropriate)

**Name:**

**Date:**

**Signature:**
